# Reduction of Endothelial Nitric Oxide Increases the Adhesiveness of Constitutive Endothelial Membrane ICAM-1 through Src-Mediated Phosphorylation

**DOI:** 10.3389/fphys.2017.01124

**Published:** 2018-01-10

**Authors:** Feng Gao, Brandon P. Lucke-Wold, Xiang Li, Aric F. Logsdon, Li-Chong Xu, Sulei Xu, Kyle B. LaPenna, Huaqi Wang, M. A. Hassan Talukder, Christopher A. Siedlecki, Jason D. Huber, Charles L. Rosen, Pingnian He

**Affiliations:** ^1^Department of Cellular and Molecular Physiology, College of Medicine, Pennsylvania State University, Hershey, PA, United States; ^2^Department of Neurosurgery, West Virginia University School of Medicine, Morgantown, WV, United States; ^3^The Center for Neuroscience, West Virginia University School of Medicine, Morgantown, WV, United States; ^4^Department of Basic Pharmaceutical Sciences, West Virginia University School of Pharmacy, Morgantown, WV, United States; ^5^Department of Surgery, College of Medicine, Pennsylvania State University, Hershey, PA, United States; ^6^Respiratory Department, The First Affiliated Hospital of Zhengzhou University, Zhengzhou, China

**Keywords:** constitutive ICAM-1, ICAM-1-mediated leukocyte adhesion, basal nitric oxide, conformational change of ICAM-1, atomic force microscopy

## Abstract

Nitric oxide (NO) is a known anti-adhesive molecule that prevents platelet aggregation and leukocyte adhesion to endothelial cells (ECs). The mechanism has been attributed to its role in the regulation of adhesion molecules on leukocytes and the adhesive properties of platelets. Our previous study conducted in rat venules found that reduction of EC basal NO synthesis caused EC ICAM-1-mediated firm adhesion of leukocytes within 10–30 min. This quick response occurred in the absence of alterations of adhesion molecules on leukocytes and also opposes the classical pattern of ICAM-1-mediated leukocyte adhesion that requires protein synthesis and occurs hours after stimulation. The objective of this study is to investigate the underlying mechanisms of reduced basal NO-induced EC-mediated rapid leukocyte adhesion observed in intact microvessels. The relative levels of ICAM-1 at different cell regions and their activation status were determined with cellular fractionation and western blot using cultured human umbilical vein ECs. ICAM-1 adhesiveness was determined by immunoprecipitation in non-denatured proteins to assess the changes in ICAM-1 binding to its inhibitory antibody, mAb1A29, and antibody against total ICAM-1 with and without NO reduction. The adhesion strength of EC ICAM-1 was assessed by atomic force microscopy (AFM) on live cells. Results showed that reduction of EC basal NO caused by the application of caveolin-1 scaffolding domain (AP-CAV) or NOS inhibitor, L-NMMA, for 30 min significantly increased phosphorylated ICAM-1 and its binding to mAb1A29 in the absence of altered ICAM-1 expression and its distribution at subcellular regions. The Src inhibitor, PP1, inhibited NO reduction-induced increases in ICAM-1 phosphorylation and adhesive binding. AFM detected significant increases in the binding force between AP-CAV-treated ECs and mAb1A29-coated probes. These results demonstrated that reduced EC basal NO lead to a rapid increase in ICAM-1 adhesive binding via Src-mediated phosphorylation without *de novo* protein synthesis and translocation. This study suggests that a NO-dependent conformational change of constitutive EC membrane ICAM-1 might be the mechanism of rapid ICAM-1 dependent leukocyte adhesion observed *in vivo*. This new mechanistic insight provides a better understanding of EC/leukocyte interaction-mediated vascular inflammation under many disease conditions that encounter reduced basal NO in the circulation system.

## Introduction

Nitric oxide (NO), in addition to being a potent vasodilator, has been recognized to play dual roles in inflammation. Excessive (pathogenic) NO production induced by inflammatory conditions/mediators contributes to an increase in microvascular permeability and plays a pro-inflammatory role (Moncada and Higgs, 1993; Yuan et al., 1993; Mayhan, 1994; Wu et al., 1996; He et al., 1997; Zhu and He, 2005; Hatakeyama et al., 2006; Sánchez et al., 2008; Zhou and He, 2011b). In contrast, physiologic (basal/constitutive) production of NO has anti-adhesive and anti-inflammatory properties (Furchgott and Vanhoutte, 1989; Ignarro, 1989; Moncada, 1992; Moncada and Higgs, 1993) and plays a critical role in preventing leukocyte adhesion (Kubes et al., 1991; Tsao et al., 1994) and the adhesion and aggregation of platelets (Azuma et al., 1986; Radomski et al., 1987a,c, 1990). Studies have shown that inhibition of basal NO increased leukocyte and platelet adhesion to cultured endothelial monolayers or vascular walls (Radomski et al., 1987b; Kubes et al., 1991, 1993; Ma et al., 1993; Tsao et al., 1994). The mechanisms were basically attributed to the direct or indirect roles of NO/cGMP in the regulation of adhesion molecules on leukocytes and the adhesive properties of platelets (Kubes et al., 1991, 1993; Ma et al., 1993; Tsao et al., 1994). A few studies indicated that the reduction of NO by pharmacological approach, genetic deletion of eNOS, or the over expression of caveolin-1 increased the adhesiveness of endothelial cells (ECs) and EC-leukocyte interaction (Radomski et al., 1987b; Ma et al., 1993; Tsao et al., 1994; Fernández-Hernando et al., 2009, 2010; Atochin and Huang, 2010; Ponnuswamy et al., 2012), but the mechanisms are still unknown. Reduced basal NO synthesis or NO bioavailability occurred in various disease conditions including hypertension, diabetes, hypercholesterolemia, and reperfusion injury, and was directly linked to endothelial dysfunction and vascular inflammation (Dinerman et al., 1993; Moncada and Higgs, 1993, 2006; Potenza et al., 2009). A better understanding of the underlying mechanisms of the anti-adhesive roles of NO will further benefit the knowledge translation to clinical applications.

**Figure 1:**
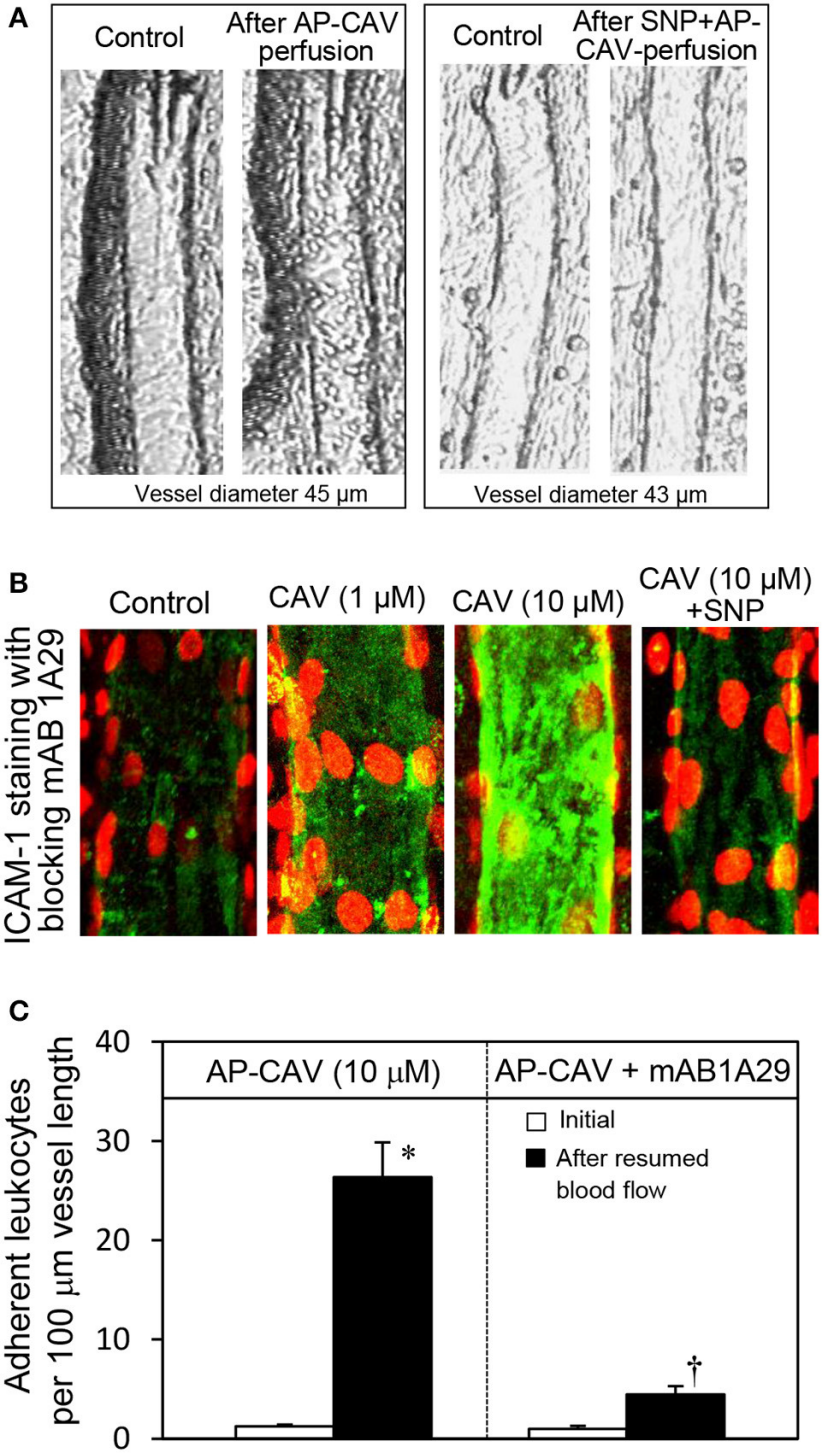
Perfusion of rat mesenteric venules with AP-CAV induced basal NO-dependent ICAM-1 mediated leukocyte adhesion. Intact venules were perfused by AP-CAV for 30 min followed by resuming blood flow in the same vessel for 10 min. Leukocyte adhesion was quantified when each vessel was recannulated with BSA-Ringer solution (Xu et al., 2013). **(A)** Video images of a perfused venule under control conditions and after AP-CAV (10 μM)-induced leukocyte adhesion, and the administration of a NO donor, sodium nitroprusside (SNP), in both perfusate (10 μM) and superfusate (20 μM) abolished AP-CAV-induced leukocyte adhesion. **(B)** AP-CAV induced dose-dependent increases in EC ICAM-1 binding to its blocking antibody mAb1A29. Confocal images of mAb1A29 (green) and vascular cell nuclei (red) immunofluorescence co-staining under control conditions, after AP-CAV perfusion, and after adding SNP to AP-CAV perfused vessels. **(C)** Perfusion of vessels with ICAM-1 inhibitory antibody, mAb1A29, significantly attenuated AP-CAV induced leukocyte adhesion (*n* = 5 per group). ^*^ and † indicate a significant increase and decrease from the control, respectively (modified from Xu et al., 2013 and used by original authors).

Our previous study conducted in intact rat venules showed that reduced endothelial basal NO synthesis caused by the application of caveolin-1 scaffolding domain (AP-CAV) or NOS inhibitor, L-NMMA, induces a large number of firmly adhered leukocytes in about 10-30 min, which was blocked by ICAM-1 blocking antibody, mAb1A29, and abolished by a NO donor, indicating a NO-dependent regulation of endothelial ICAM-1 (Xu et al., 2013). The key observations derived in intact microvessel studies (Xu et al., 2013) are presented in Figure 1. However, the intact microvessel studies could not provide mechanistic evidence addressing how reduced basal NO increases ICAM-1-mediated early phase leukocyte adhesion and whether the increased leukocyte adhesion is caused by increased quantity of membrane expression of ICAM-1 through translocation or purely due to the quality change of constitutively expressed ICAM-1, namely by increasing its adhesion capacity. This study conducted in cultured human umbilical vein endothelial cells (HUVECs) is the extension of our intact microvessel studies and aims to provide the mechanistic insight for this important *in vivo* observation at cellular and protein levels. We quantitatively measured real-time endothelial NO production in the absence and presence of AP-CAV, an endogenous eNOS inhibitor, and L-NMMA, and assessed the concomitant changes in sub-cellular quantity of ICAM-1, adhesive binding avidity of ICAM-1, as well as the signaling pathways involved in the activation status of ICAM-1.

## Materials and methods

### Endothelial cell culture and treatments

Primary human umbilical endothelial cells (HUVECs), endothelial growth media (EGM), and supplements were purchased from Lonza (CC-3122 and CC-4133, Walkersville, MD). HUVECs were seeded at a density of 2.5 x 10^3^ cells per cm^2^ and cultured in EGM with supplements containing bovine brain extract, human epidermal growth factor, fetal bovine serum, hydrocortisone, ascorbic Acid, GA-1000 (Gentamicin, Amphotericin B), and heparin. The cell culture was performed in a humidified atmosphere of 5% CO_2_ at 37°C. HUVECs were split routinely when they reached 90% confluence and used for experiments within 5 passages after purchasing. Cells were treated with AP-CAV, the fusion peptide of CAV scaffolding domain with AP, the Antennapedia internalization sequence from Drosophila Antennapedia homeodomain (synthesized by Tufts University), PP1, [(4-Amino-5-(methylphenyl)-7-(t-butyl)pyrazolo-(3,4-d)pyrimidine, Sigma], and/or N(Ω)-monomethyl-L-arginine acetate salt (L-NMMA, Sigma) for 30 min or 6 h at a concentration of 10 μM. In some experiments, PP1 was added 30 min prior to AP-CAV peptide treatment. ECs cultured in petri dish were used for western blot, and microfluidic devices were used for NO measurements. The stock solutions of AP-CAV peptide, PP1, and L-NMMA were prepared in 100% DMSO, and the final solution of each agent was prepared by >1:1,000 dilution of the stock with EC culture medium.

### NO measurements and fluorescence imaging in HUVECs cultured in microfluidic channels

We recently demonstrated that ECs develop well-formed junctions when grown with continuous flow in the microchannels, and their intracellular calcium and nitric oxide responses to ATP were similar to those observed in intact microvessels (Li et al., 2015). In this study, cultured EC-formed microvessel networks were used to measure changes in EC basal NO production rate before and after AP-CAV or L-NMMA was applied to the vessel lumen. The methods have been described in detail (Zhou and He, 2011a; Li et al., 2015; Xu et al., 2016). In brief, HUVECs were seeded into polydimethylsiloxane (PDMS) microfluidic microchannel network device and cultured under continuous perfusion with wall shear stress at 1.0~2.0 dyne/cm^2^ within the network. Experiments were conducted after ECs reached confluency (4~5 days of perfusion). DAF-2 DA, a membrane-permeable fluorescent indicator for NO (Sigma), was used to measure EC NO. Since the chemical conversion of DAF-2 in reaction of NO is irreversible, the changes of DAF-2 fluorescence (FI_DAF_) represents the cumulative NO production. The magnitude change of FI is time and rate dependent. So the rate change of FI_DAF_, df/dt, or NO production rate, is the most appropriate way to report the DAF-2 results. In this study, NO production rate was reported, which was derived from FI_DAF_ profile in microvessel networks. Leica TCS SP8 confocal system and Leica × 25 objective (NA: 0.95) were used for fluorescence imaging. An optically pumped solid-state laser at 488 nm with 0.08% power was used for excitation, and a HyD detector was used for the collection of the 510–545 nm emission. In each experiment, the microvessel network was continuously perfused with albumin-Ringer solution containing DAF-2 DA (5 μM). After the DAF-2 loading reached steady state (about 40 min perfusion), image stacks (xyz) were collected at 1 min interval using 1,024 × 1,024 scan format and 0.5 μm z-step. In each experiment, the images of FI_DAF_ were collected under control conditions first for calculation of basal NO production rate and followed by the addition of 10 μM of AP-CAV or L-NMMA. Sodium nitroprusside (SNP, 50 mM, Sigma), a NO donor, was perfused at the end of the experiment to verify the sufficiency of DAF-2 within ECs and the specificity of DAF-2 response to NO. Image analysis and NO production rate calculations have been described in detail (Zhou and He, 2011a).

### Subcellular fractionation and western blot analysis

To investigate ICAM-1 expression at different subcellular compartments, control and treated HUVECs were lysed as whole cell, or into cytosolic and membrane fractions using a standard Cell Fractionation Kit (ab109719, Abcam) for ICAM-1 expression studies. All collected proteins were centrifuged at 12,000 × g for 15 min at 4°C, and the protein concentration was measured by a BCA assay (Pierce). For each set of experiments, equal amounts of protein (20~25 μg/well) were loaded to 10% SDS-PAGE gels (Bio-Rad Laboratories) for electrophoresis in a Bio-Rad mini blot system (Life Technologies) at constant voltage of 90V, and transferred to nitrocellulose membranes (Life Technologies) at constant current of 100 mA for 2 h at 4°C. The membranes were then incubated at room temperature in Odyssey blocking buffer (LiCor BioSciences) for 1 h, followed by incubations with primary antibodies [1:1,000 for anti-VE-Cadherin (Cat # 2500, Cell Signaling), anti ICAM-1 (1:1,000, Cat # 4915S, Cell Signaling or Cat # 554966, BD Pharmingen), and anti-β-actin (Cat # 8457, Cell Signaling), 1:500 for anti-p-ICAM-1 (phospho Y512, ab51033, Abcam)] at 4°C overnight, and secondary antibodies [1:10,000 for IRDye800-conjugated anti-mouse and anti-rabbit antibodies (Cat # 925-68071 and 925-32211, LiCor BioSciences)] at room temperature for 1 h. Images were visualized using a LiCor Odyssey Scanner and the bands were quantified using Image Studio software (LiCor BioSciences). All of the values were presented as ratio to the reference protein in the same lane.

### Immunoprecipitation analysis

To investigate the mechanisms involved in increased adhesiveness of ECs in response to reduced NO, immunoprecipitation analysis was conducted in non-denatured proteins. HUVECs under control conditions or after treatments were collected with non-denaturing lysis buffer (20 mM Tris HCl pH 8, 137 mM NaCl, 1% Triton X-100, 2 mM EDTA, and protease inhibitors) for immunoprecipitation analysis. All of the subsequent procedures were performed according to manufacturer's protocol. Briefly, protein G Dynabeads IP kit was used (10007D, Life Technologies) for binding of ICAM-1 blocking antibody (mAb1A29, 1:1,000, Cat # 554966, BD Pharmingen) and antibody against total ICAM-1 (1:1,000, Cat # 4915S, Cell Signaling) to magnetic beads, immunoprecipitation of antigen, and elution of Ab/Ag complex. Supernatants collected after elution were transferred to clean tubes, and the immunoprecipitation samples were then prepared for gel loading, electrophoresis, membrane transfer, blocking, and incubations with ICAM-1 blocking antibody, total ICAM-1 antibody, and anti-GAPDH antibody (1:3,000, ab9485, Abcam), as described above for the western blot.

### AFM measurements of cell membrane ICAM-1 binding force

The adhesive binding of cell membrane ICAM-1 to the blocking antibody mAb1A29 was measured by AFM in ECs cultured in petri dish with slight modifications of previously described procedures (Xu and Siedlecki, 2010; Ozdemir et al., 2013). Briefly, silicon nitride AFM probes (DNP, Bruker, spring constant 0.06 N/m) were first cleaned with acetone for 15 min, followed by a 30 min of air plasma treatment, and then incubation with 1% aminopropyltriethoxysilane solution in ethanol for 1 h. After three times of rinse with deionized (DI) water, the probes were incubated with 10% glutaraldehyde solution for 1 h to provide a cross linking site. After three times of additional washing with DI water, probes were then incubated with ICAM-1 blocking antibody (25 μg/ml, mAb1A29) for 1 h and stored in phosphate-buffered saline (PBS) until use within 2 days. HUVECs were seeded on collagen-coated (0.5 mg/ml) glass coverslips. Upon 80% confluence, the cells were treated accordingly to the experimental protocol, and the coverslips were then attached on a metal disc platform where the cells were maintained with serum free EC culture medium. The force measurement was carried out in aqueous solution with a fluid cell containing HUVEC medium on a multimode AFM with a Nanoscope IIIa control system (version 5.31r1, Veeco Instruments, Santa Barbara, CA). ECs, AFM force curves (over 60 measurements in each group) were randomly acquired from cultured EC surface (three separated areas at size of 60 μm × 60 μm each) in the force mode at a vertical scan rate of 1 Hz and ramp size of 25,00 nm. The trigger mode was set with a deflection threshold of 50 nm. The retraction force curves were extracted for the calculation of binding force, which was defined as the product of spring constant and maximum deflection of cantilever during the probe separation from sample surface.

### Statistical analysis

All numerical data were presented as mean ± SE for each group, except where noted otherwise. Paired *t*-test was used for paired data analysis. Unpaired *t*-test was used between two groups. One-Way ANOVA with *post-hoc* Tukey test was used to compare the differences between groups of three or more. Kolmogorov-Smirnov test was performed to compare the distribution of atomic force between control and AP-CAV-1 treated groups and presented as median with 95% confidence interval. A probability value of *P* < 0.05 was considered statistically significant.

## Results

### AP-CAV inhibits basal NO production in HUVECs cultured in microfluidic channels

**Figure 2:**
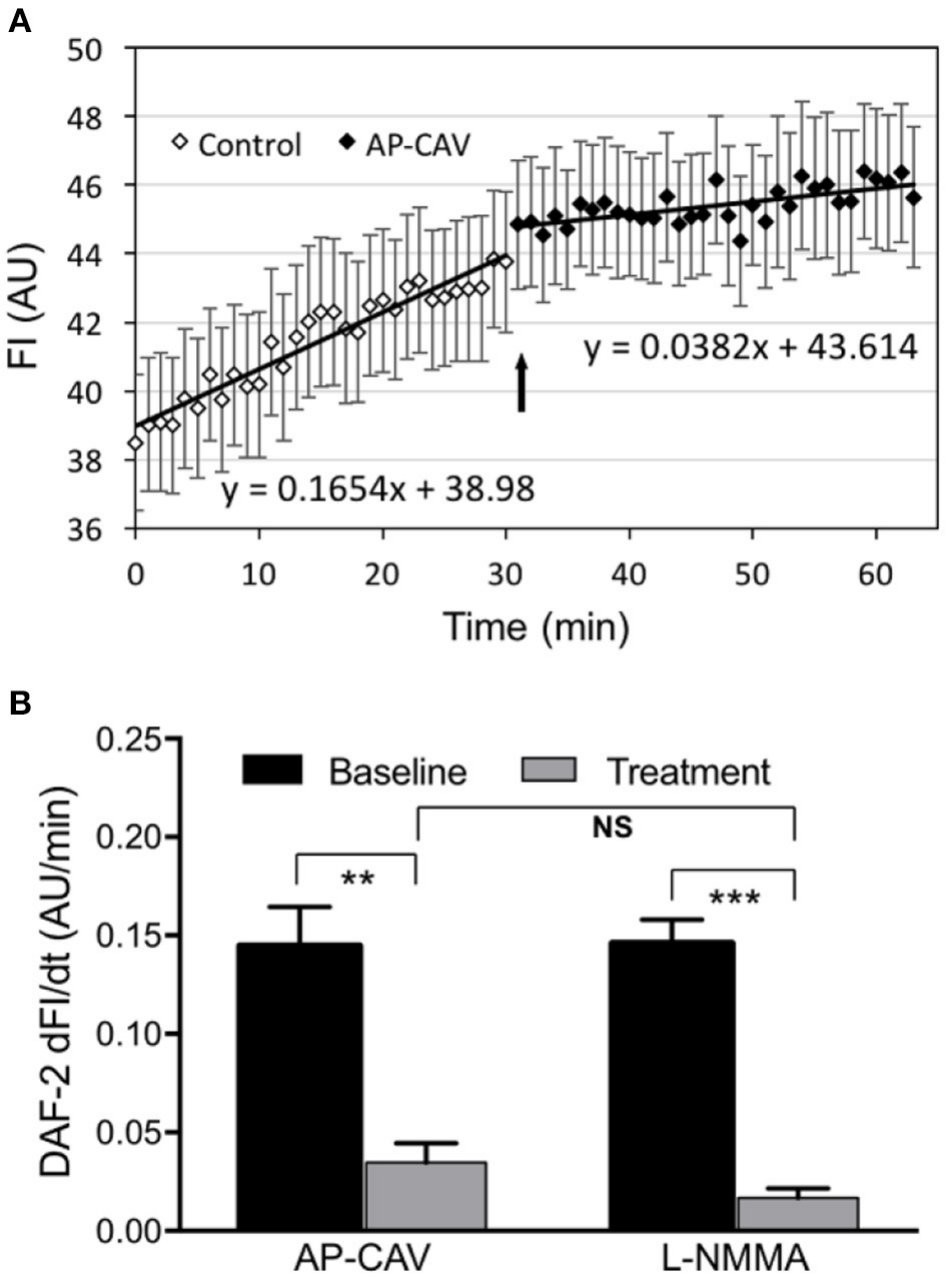
AP-CAV or NOS inhibitor reduced basal NO production in HUVECs cultured in microchannels of microfluidic devices. **(A)** A representative experiment showing the time-dependent changes of DAF-2 fluorescence (FI_DAF_) before and after AP-CAV (10 μM) application. Arrow indicates the time point when AP-CAV was perfused into the microfluidic device. **(B)** Summary of NO production rate derived from FI_DAF_ profiles (*n* = 4 per group). ^**^P < 0.005, ^***^P < 0.0005. NS indicates no significant differences.

The effect of AP-CAV on basal endothelial NO production was evaluated in four microfluidic devices with confluent HUVECs in the microchannel network. The representative profile of DAF-2 fluorescent intensity (FI_DAF_) from ECs of microfluidic channels was presented in Figure 2A. With albumin-Ringer perfusion, FI_DAF_ showed a slow increase at a constant rate of 0.14 ± 0.020 AU/min, which represents the rate of EC basal NO production. When AP-CAV (10 μM) was added to the perfusion of microfluidic channels, the rate of the FI_DAF_ increase significantly reduced from the basal level to a mean value of 0.04 ± 0.017 AU/min. We also compared the inhibitory effect of AP-CAV on basal NO with that of a NOS inhibitor, L-NMMA. In another set of four HUVEC microfluidic channels, the mean rate of FI_DAF_ increase in the presence of L-NMMA (10 μM) was significantly reduced from the basal level of 0.15 ± 0.012 AU/min to 0.02 ± 0.005 AU/min. Results are summarized in Figure 2B. Both the basal NO production rate and the magnitude of basal NO reduction by AP-CAV or L-NMMA measured in cultured HUVEC microchannels were similar to those measured in individually perfused intact rat microvessels (Zhou and He, 2011a; Xu et al., 2013).

### AP-CAV-induced basal NO reduction does not alter ICAM-1 expression and constitutive ICAM-1 subcellular distribution

**Figure 3:**
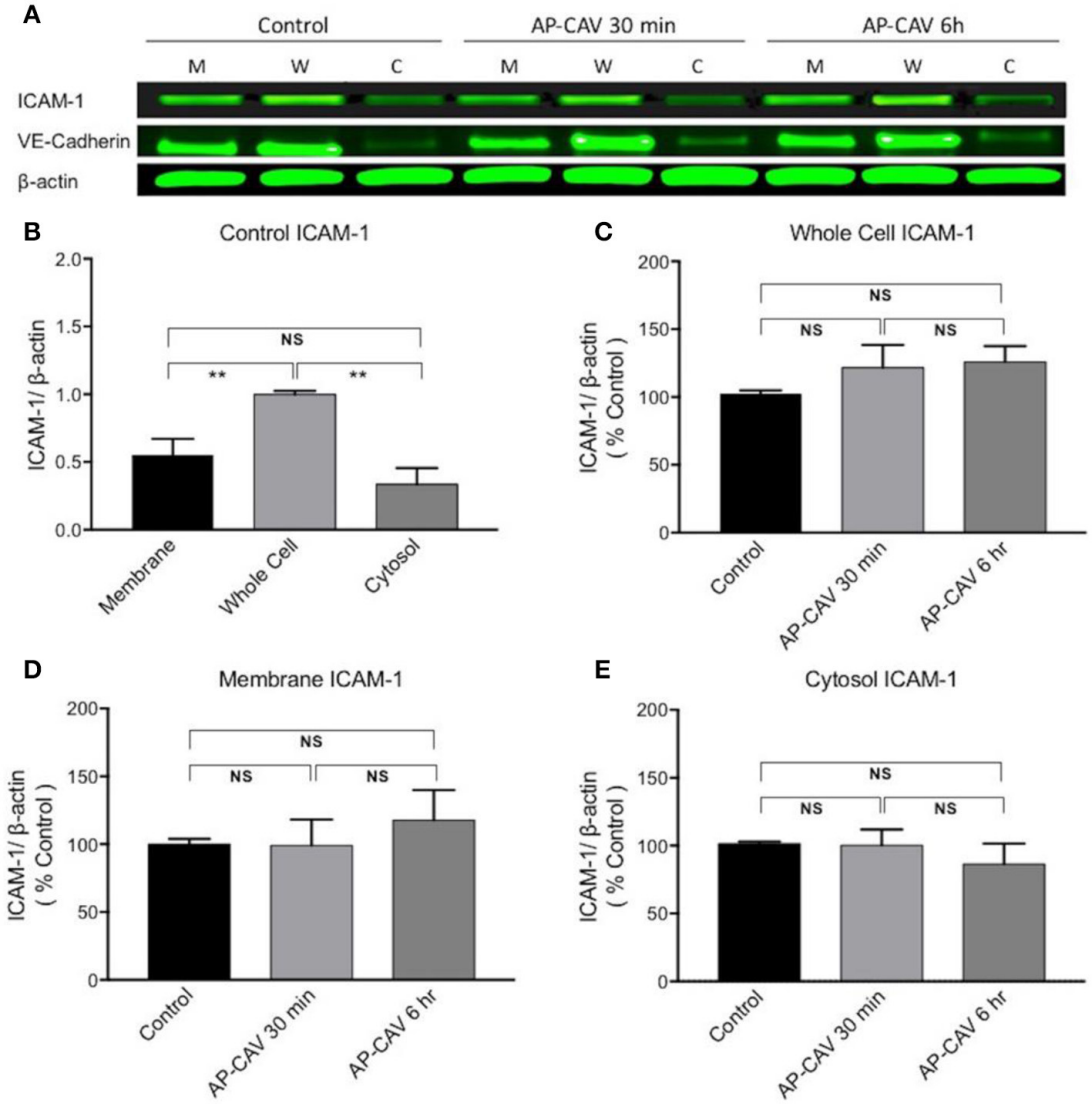
AP-CAV application shows no effect on ICAM-1 expression at whole cell and subcellular fractions of HUVECs. **(A)** Western blot showed no significant changes in ICAM-1 expression in whole cell (W), membrane (M), and cytosol (C) of HUVECs after AP-CAV (10 μM) treatment for 30 min or 6 h, respectively. VE-Cadherin, a membrane bound protein, that was only evident in the membrane and whole cell validated the cell fractionation procedure. **(B)** Summary of ICAM-1 expression in whole cell and subcellular fractions of HUVECs under control conditions. **(C–E)** Result summary showing no significant changes of ICAM-1 expression after AP-CAV (10 μM) treatment for 30 min or 6 h in whole cell and subcellular locations. *n* = 3 per group. ^**^P < 0.005; NS indicates no significant differences.

Our previous study observed significant increases in firmly adhered leukocytes to rat venules after perfusion of vessels with AP-CAV for about 30 min, and pre-perfusing vessels with ICAM-1 blocking antibody, mAb1A29, prevented such adhesion, indicating that the adhesion process was mediated by AP-CAV-induced changes in endothelial ICAM-1 (Xu et al., 2013). The short timing of AP-CAV-induced leukocyte adhesion basically ruled out the possibility of *de novo* ICAM-1 synthesis, but the intact microvessel study could not distinguish if AP-CAV-induced ICAM-1-mediated leukocyte adhesion was the result of increased membrane ICAM-1 through its translocation from cytosol or purely an increase in the binding capacity and/or binding affinity of constitutive membrane ICAM-1 to leukocytes. In this study, we first evaluated ICAM-1 expressions at different fractions of ECs by western blot analysis following 30 min and 6 h treatments with AP-CAV peptide. Under control conditions, the mean ICAM-1 expression in the cell membrane is higher than that in the cytosol, but not statistically different. After AP-CAV (10 μM) treatments, we observed no significant changes in ICAM-1 expression from that of the control in both whole cell and subcellular fractions (Figure 3, *P* > 0.05, *n* = 3 per group), indicating that AP-CAV has no effect on either *de novo* synthesis or translocation of EC ICAM-1. The cellular fractionation process was validated by the differentiated distributions of EC membrane protein VE-cadherin that showed dominated expressions in the membrane fraction and minimum in cytosol (Figure 3A).

### AP-CAV increases ICAM-1 binding strength to its blocking antibody: binding force measurements

**Figure 4:**
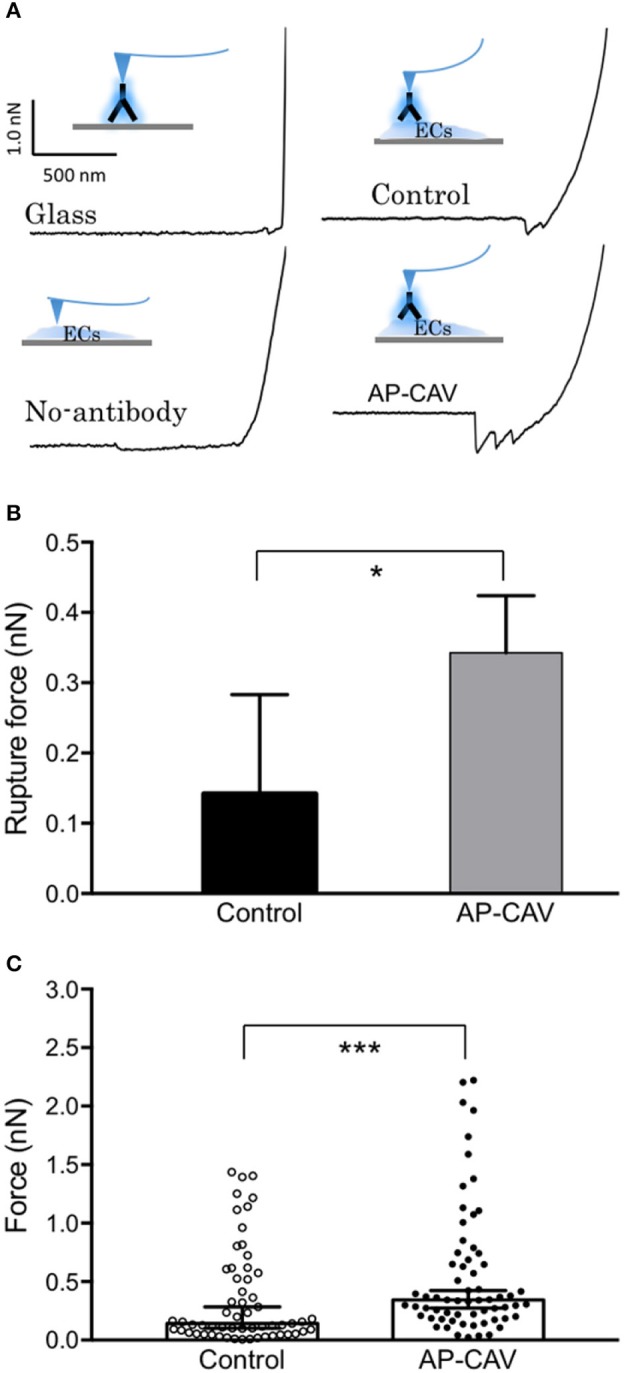
Reduced endothelial basal NO by AP-CAV increases the binding strength of live cell membrane ICAM-1 to its blocking antibody. **(A)** Force curves differ between groups (contacting surfaces with and without cultured ECs, probes with and without coated ICAM-1 blocking antibody, and cultured ECs with and without exposure to AP-CAV). The curve with a gradual decrease indicates the adhesive interaction between ICAM-1 blocking antibody (on probe) and ICAM-1 on EC membrane. **(B)** Result summary showing that 30 min application of AP-CAV (10 μM) significantly increased EC ICAM-1 adhesive strength. Values shown are median with 95% confidence interval. **C)** The distribution of each measured force value in control and AP-CAV treated cells. More than 60 measurements were acquired in three separate cell surface areas (60 μm × 60 μm each) in each cell group. Kolmogorov-Smirnov test was performed to compare the distribution differences between two groups. ^*^P < 0.05, ^***^P < 0.0005.

The western blot analysis with no AP-CAV-induced changes in ICAM-1 expression in all subcellular compartments suggested that reduced basal NO by AP-CAV might increase the adhesiveness of constitutive ICAM-1 in EC (Xu et al., 2013). To test this possibility, AFM was used to measure the binding strength of EC ICAM-1 to its blocking antibody mAb1A29. The representative retraction force curves on various surfaces are illustrated in Figure 4A. When ICAM-1 antibody coated AFM probes compressed and then retracted from the EC membrane (Figure 4A, control), the force curves showed a gradually decreased value compared to the steeper curve slope on the glass surface (Figure 4A, glass). The steepness of force curve was used to determine whether the probe landed on the cell membrane surface or not. When a probe without antibody coating was used for detection, there was no abrupt shift as the probe separated from the cell surface, indicating no binding event occurred between probe and cell membrane. In contrast, ICAM-1 antibody coated probe displayed the saw-like force curve, indicating multiple debonding events simultaneously occurred in the separation region when the probe retracted from cell membranes. Results showed that AP-CAV (10 μM)-treated ECs markedly increased the debonding force compared to that measured on control cells. The median binding strengths in control and AP-CAV treated cells were 0.14 nN and 0.34 nN, respectively (Figure 4B, *P* < 0.05, *n* > 60). Figure 4C shows significantly different distributions of binding strength measured in control and AP-CAV treated cells (*P* < 0.0005), indicating an increased binding strength between EC ICAM-1 and its blocking antibody in AP-CAV-treated cells.

### Reduction of basal NO caused a quick increase in EC ICAM-1 adhesive binding avidity without altering ICAM-1 expression: immunoprecipitation assay in non-denatured proteins

**Figure 5:**
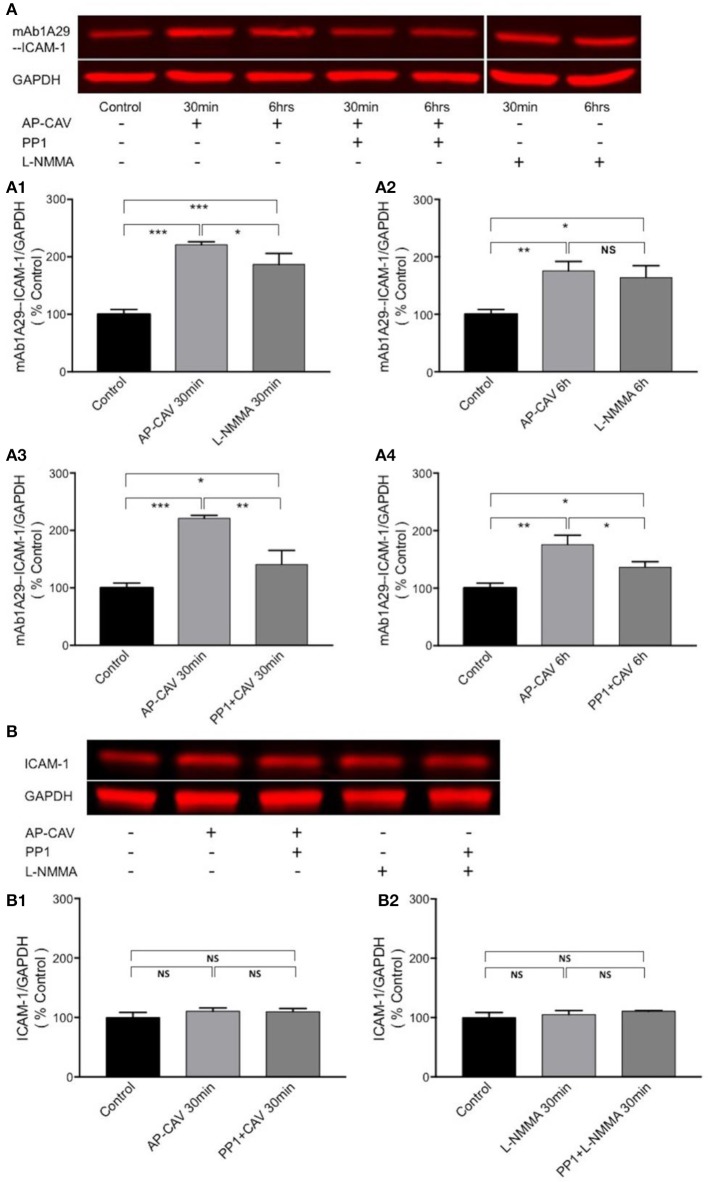
Immuno-precipitation assay conducted in non-denatured proteins showing that reduction of basal NO increases the adhesiveness of EC constitutive ICAM-1 via Src-dependent pathway. **(A)** Reduced basal NO increased EC ICAM-1 binding to its inhibitory antibody. mAb1A29. The application of AP-CAV (10 μM) or L-NMMA (100 μM) for 30 min **(A1)** or 6 h **(A2)** significantly increased ICAM-1 binding to its blocking antibody. Pre-treatment of ECs with Src kinase inhibitor, PP1 (10 μM), significantly attenuated the effect of AP-CAV, indicating the involvement of Src signaling **(A3,A4)**. **(B)** In contrast to the results shown in A, reduction of basal NO did not change ICAM-1 binding to antibody against total ICAM-1, indicating no changes in ICAM-1 expression. AP-CAV **(B1)** or L-NMMA **(B2)** treatment for 30 min with or without PP1 did not alter ICAM-1 binding to antibody against total ICAM-1. *n* = 3 for all IP and western blot assays. ^*^P < 0.05, ^**^P < 0.005, ^***^P < 0.0005, NS indicates no significant differences.

To investigate if the AP-CAV-mediated increases in EC adhesiveness is directly related to increased ICAM-1 binding to its counter ligand, immuno-precipitation assays were performed in non-denatured cell lysate to assess the changes in ICAM-1 binding to its blocking antibody, mAb1A29, and compared with its binding to antibody against total ICAM-1 under identical experimental conditions. Data shown in Figure 5 demonstrated that both 30 min and 6 h of AP-CAV (10 μM) treatment increased bound mAb1A29-ICAM-1 compared to the untreated control group (*n* = 3 per group). To further confirm reduced basal NO to be the cause of AP-CAV-mediated increase in ICAM-1-mAb1A29 complex, NOS inhibitor, L-NMMA (100 μM), was applied to cells for 30 min and 6 h, respectively. The results were comparable to that of AP-CAV (n = 3 per group). We also examined the involvement of Src signaling in such effects. A Src kinase inhibitor, PP1 (10 μM), was applied to cells before AP-CAV treatment. As shown in Figures 5A3,A4, pre-treatment of cells with PP1 significantly attenuated AP-CAV-induced increases in ICAM-1-mAb1A29 complex (*n* = 3 per group). The ICAM-1 binding to antibody against total ICAM-1 was also assessed under identical experimental conditions to those using mAb1A29. In contrast to the results of mAb1A29, both AP-CAV and L-NMMA treatments showed no effect on ICAM-1-antibody for total ICAM-1 binding compared to the control (Figure 5B). These results indicate that reduction of basal NO increased its binding to its counter ligand without changing its protein expression, and this action is mediated by Src signaling.

### AP-CAV-mediated basal NO reduction increases the adhesiveness of ICAM-1 via Src-mediated ICAM-1 phosphorylation

**Figure 6:**
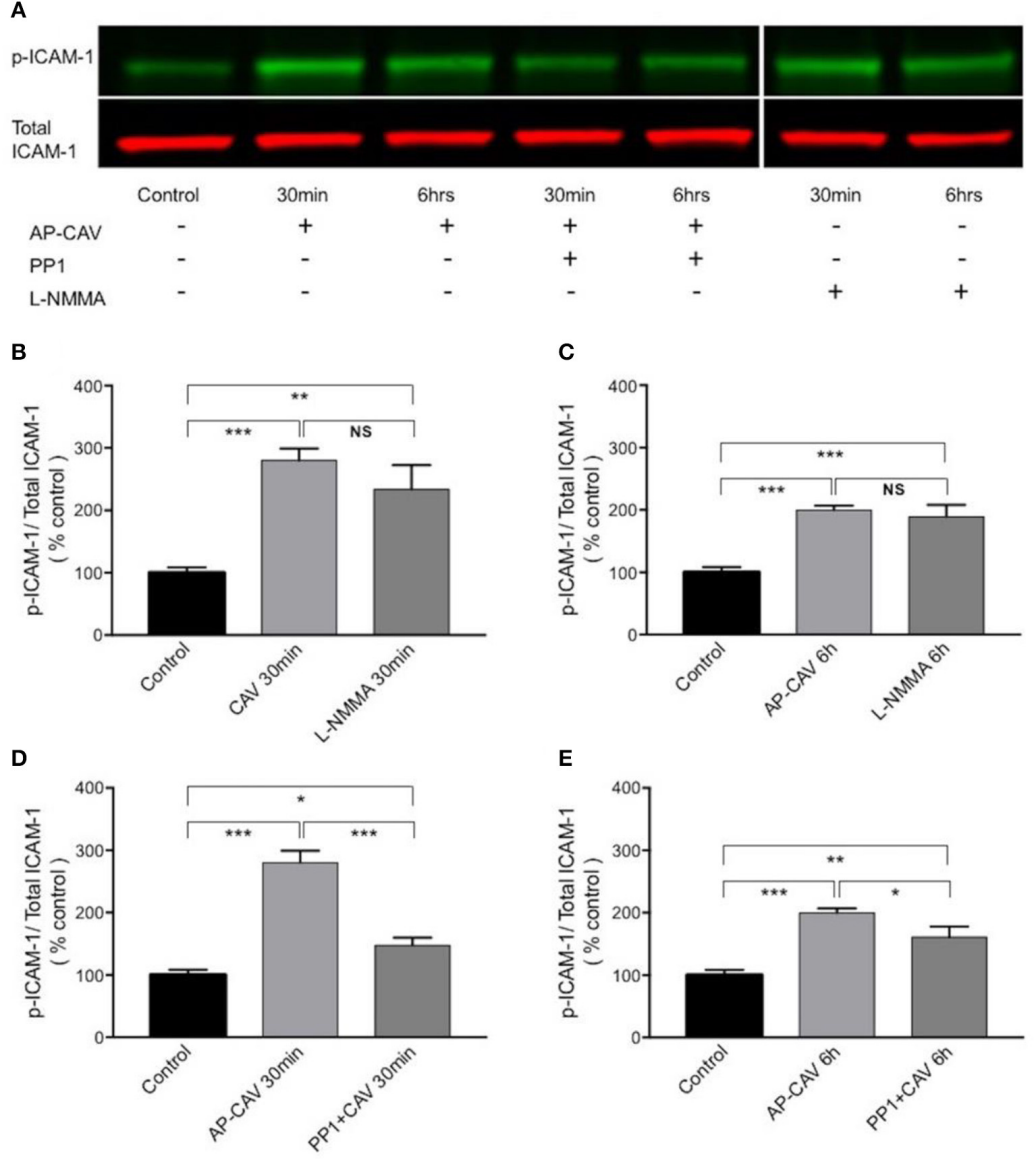
The increased adhesiveness of EC ICAM-1 induced by basal NO reduction is associated with Src signaling-mediated phosphorylation of constitutive ICAM-1. **(A)** Western blot showing phosphorylated ICAM-1 (p-ICAM-1) relative to total ICAM-1 significantly increased after HUVECs were exposed to AP-CAV (10 μM) or L-NMMA (100 μM) for 30 min **(B)**, or 6 h **(C)**. Pre-exposure cells to 10 μM PP1 significantly attenuated the effect **(D,E)**. *n* = 3 for western blot. ^*^P < 0.05, ^**^P < 0.005, ^***^P < 0.0005, NS indicates no significant differences.

**Figure 7:**
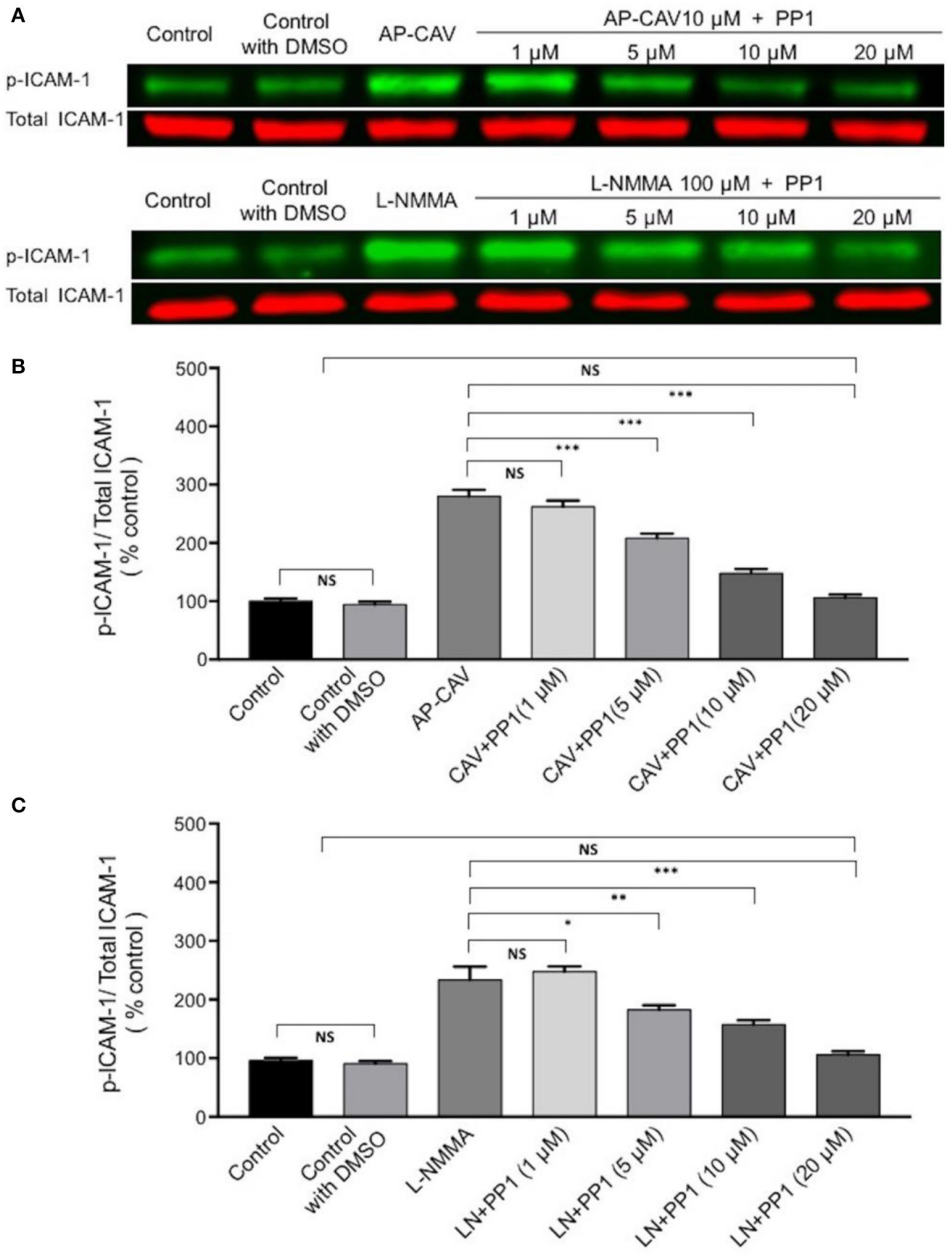
The role of Src signaling in NO-dependent phosphorylation of constitutive ICAM-1. **(A)** Western blot shows dose-dependent inhibition of phosphorylated ICAM-1 (p-ICAM-1) relative to total ICAM-1 by Src kinase inhibitor, PP1, after ECs were exposed to AP-CAV or L-NMMA. **(B)** Application of AP-CAV (10 μM) for 30 min significantly increased p-ICAM-1. PP1 at 5, 10, or 20 μM showed graded inhibition of increased p-ICAM-1. **(C)** L-NMMA (100 μM)-induced increases in p-ICAM-1 was inhibited by PP1 in a manner similar to that of AP-CAV. *n* = 3 for all experiments. ^*^P < 0.05, ^**^P < 0.005, ^***^P < 0.0005, NS indicates no significant differences.

The findings that Src kinase inhibitor significantly attenuated AP-CAV-induced increase in ICAM-1/mAb1A29 binding (Figure 5A) lead us to examine whether Src-mediated phosphorylation of ICAM-1 is involved. We determined the expression levels of phosphorylated ICAM-1 (p-ICAM-1) relative to the total ICAM-1 in the absence and presence of AP-CAV (10 μM). Figure 6A shows the representative results of western blot. The summary results shown in Figures 6B,C demonstrated that the application of AP-CAV (10 μM) significantly increased p-ICAM-1 in HUVECs compared to untreated control group (*n* = 3 per group). The similar results observed with LNMMA application further support the reduced basal NO by AP-CAV to be the mechanism of ICAM-1 activation. Figures 6D,E show that pre-treatment of the cells with PP1 (10 μM, 30 min) significantly reduced AP-CAV-induced increases in p-ICAM-1 (*n* = 3 per group). The role of Src signaling in ICAM-1 activation was further evaluated with Src kinase inhibitor at different dosages. Figure 7 shows that Src kinase inhibitor, PP1, at concentrations between 5 and 20 μM showed graded inhibition of AP-CAV- and L-NMMA-induced increases in p-ICAM-1 (*n* = 3 per group). The p-ICAM-1 levels with 20 μM of PP1 treatment were not significantly different from that of the control, indicating a complete inhibition. These results indicate that reduced basal NO activates constitutive ICAM-1 via Src-mediated phosphorylation.

## Discussion

This study revealed a novel mechanism of NO-dependent changes in EC adhesiveness that had been observed in intact microvessels (Xu et al., 2013). The major new findings are that reduction of endothelial basal NO by direct inhibition of endothelial eNOS with AP-CAV or L-NMMA induced a rapid activation of constitutively expressed endothelial ICAM-1 via Src-mediated phosphorylation, resulting in increases in adhesive binding of ICAM-1. The identified mechanism explained our previous observation that reduced endothelial basal NO quickly induces ICAM-1-mediated firm adhesion of leukocytes to rat venules (Xu et al., 2013). Most importantly, the reduction of basal NO-induced increases in ICAM-1 adhesiveness occurred in the absence of *de novo* protein synthesis and translocation, suggesting a NO-dependent regulation of the adhesive binding avidity of constitutively expressed endothelial ICAM-1. These findings provide new insights into the mechanisms whereby reduced basal NO increases the adhesiveness of endothelial cells and contributes to early phase leukocyte adhesion to microvessels.

### Inhibition of basal NO synthesis in cultured HUVECs

In this study the inhibition of endothelial basal NO was achieved by the application of an eNOS endogenous inhibitor, AP-CAV, and confirmed by a NOS inhibitor, L-NMMA. eNOS is constitutively expressed in the caveolae and colocalizes with CAV-1 (Feron et al., 1996; García-Cardeña et al., 1997). Under basal conditions most of the eNOS is associated with scaffolding domain of CAV-1, which tonically inhibits eNOS activation and function (Bucci et al., 2000; Fleming, 2010). In the CAV-1-null mice, eNOS activity is upregulated because of the loss of CAV-1 (Drab et al., 2001; Razani et al., 2001), and functional studies showed that CAV scaffolding domain peptide can selectively regulate signal transduction to endothelial eNOS both in intact blood vessels and cultured aortic endothelial cells (Bucci et al., 2000; Bernatchez et al., 2005). Those studies thus indicated CAV-1 as an endogenous inhibitor of eNOS. We have also demonstrated that application of AP-CAV in intact microvessels inhibits platelet activating factor (PAF)-induced NO production and NO-mediated increases in microvessel permeability (Zhu et al., 2004; Xu et al., 2013). Using a newly developed method to measure basal NO with continuous perfusion of NO indicator DAF-2 DA (Zhou and He, 2011b), we were the *first* to show that AP-CAV reduces endothelial basal NO production without affecting basal microvessel permeability, but promotes ICAM-1-mediated firm leukocyte adhesion in intact rat mesenteric venules (Xu et al., 2013). Using this NO detection technique along with an improved cell culture with microfluidic devices (Li et al., 2015; Xu et al., 2016), this study provides direct evidence that exogenously applied AP-CAV suppresses real-time basal NO production in cultured HUVECs, and these findings are consistent with our earlier study in intact microvessels (Xu et al., 2013). With validated basal NO reduction by AP-CAV or L-NMMA, we further investigated the mechanisms of reduced basal NO-induced leukocyte adhesion in intact microvessels.

### The role of basal NO in the regulation of the adhesiveness of constitutively expressed endothelial ICAM-1

The role of basal NO in preventing leukocyte-EC interaction, platelet adherence and aggregation has been well documented previously (Azuma et al., 1986; Radomski et al., 1987a,c, 1990; Kubes et al., 1991). During inflammatory process, NO has been shown to modulate the expression of adhesion molecules, leukocyte adhesion and migration, and the secretion of inflammatory mediators (Kubes et al., 1991; Ma et al., 1993; Granger and Kubes, 1994; Tsao et al., 1994). The leukocyte-EC adhesion in microcirculation has been characterized as a cascade of coordinated interactions mediated by different adhesion molecules (Carlos and Harlan, 1994; Granger and Kubes, 1994), and the firm adhesion of leukocytes in the classical pattern of leukocyte-EC interaction is mediated by increased expressions of ICAM-1 that requires transcription and translation of new proteins and occurs several hours after cytokine-mediated activation (Wertheimer et al., 1992; Carlos and Harlan, 1994; Granger and Kubes, 1994). ICAM-1 is thus considered a critical regulator of late phase leukocyte-EC interactions. In contrast to classical pattern of leukocyte adhesion, we demonstrated that reduced basal NO, caused by AP-CAV or L-NMMA, not only leads to rapid (within 10–20 min) but also firm adhesion of leukocytes in intact rat venules (Figures 1A,C; Xu et al., 2013). Immunofluorescence staining demonstrated increases in endothelial ICAM-1 binding to its blocking antibody within 30-min of AP-CAV perfusion (Figure 1B; Xu et al., 2013). Although the timing of increased ICAM-1 binding to its blocking antibody was not in the time zone of new ICAM-1 synthesis, the possibility of increased membrane ICAM-1 expression through translocation could not be ruled out in our earlier study. The regulation of endothelial adhesion molecules involves both quantitative changes in the surface expression and qualitative changes in the avidity (Carlos and Harlan, 1994; van de Stolpe and van der Saag, 1996). In this study, cellular fractionation and western blot analysis revealed that the majority of the ICAM-1 was localized in the cell membrane and there was no difference in the subcellular expression levels of ICAM-1 between untreated control and AP-CAV or L-NMMA treated cells. These findings thus demonstrated that, in our experimental conditions, AP-CAV treatment neither causes the membrane translocation nor the protein synthesis of ICAM-1.

Since no quantity change and translocation of ICAM-1 were identified following the reduction of basal endothelial NO, we then examined the role of NO in the regulation of the adhesiveness of constitutively expressed ICAM-1 on ECs. Monoclonal antibody, mAb1A29, is a specific functional inhibitory antibody against ICAM-1 adhesion sites. Its application inhibited ICAM-1-dependent leukocyte adhesion in whole animal (Morisaki et al., 1997) and in individually perfused intact microvessels (Xu et al., 2013). AFM measurements showed a significant increase in debounding force between the cell membrane ICAM-1 in AP-CAV-treated cells and mAb1A29-coated probe. The detected higher magnitude of debonding force supports that the increased adhesive binding of constitutive ICAM-1 contributes to AP-CAV-induced leukocyte adhesion observed in intact microvesels. This increased adhesive binding force was further confirmed by immunoprecipitation studies conducted in non-denatured proteins where we showed increased binding between cell membrane ICAM-1 and mAb1A29 in AP-CAV treated cells. The similar effect observed in NOS inhibitor, L-NMMA, treated cells further supports that the AP-CAV effect was caused by reduced NO. In contrast, no changes in binding complexes associated with ICAM-1 were observed in immunoprecipitation studies using antibody against total ICAM-1 under the identical experimental conditions. These results suggest that the reduced basal NO-induced increases in adhesiveness of ECs could be the result of increased adhesive binding of ICAM-1 to its counter ligands without membrane ICAM-1 translocation or net increases in protein expression. The increased adhesive binding avidity could be the result of increased adhesion binding sites due to structural conformational change or increased binding site affinity of ICAM-1. Our current study at cellular and protein levels demonstrates increases in adhesion binding avidity, but is limited in further differentiating between binding capacity, binding affinity, or a combination of both. We speculate that the increased exposure of adhesive binding sites is likely to be involved, but direct supporting evidence has to rely on structural analysis at single molecule level.

### Src signaling in reduction of basal NO-induced activation of EC constitutive ICAM-1 with increased adhesive binding avidity

Activation of cell adhesion molecules has been shown to involve multiple signaling pathways, such as non-receptor tyrosine kinases (Src), calcium, RhoA, phosphatidylinositol 3 kinase (PI3K), mitogen activated protein kinases, and NFκB (Adamson et al., 1999; Etienne-Manneville et al., 2000; Wang and Doerschuk, 2001; Lin et al., 2005; Allingham et al., 2007). Src-dependent phosphorylation of EC ICAM-1, independent of de novo protein synthesis, has been reported for TNFα-induced increase in ICAM-1 binding avidity and early phase leukocyte adhesion in both lung microvessels and cultured ECs (Liu et al., 2011). Our recent study also showed that reduced NO-mediated ICAM-1 phosphorylation and early leukocyte adhesion involves Src signaling pathway in intact microvessels (Xu et al., 2013). In this study, the Src kinase inhibitor showed inhibitory effect on both inhibitory antibody binding to ICAM-1 and p-ICAM-1 when basal NO was reduced, which strongly supports the role of Src signaling in ICAM-1 activation. More importantly the dose-dependent inhibition of ICAM-1 phosphorylation by PP1 provided further supporting evidence, especially 20 μM of PP1 basically reduced the p-ICAM-1 level insignificant to that of the control, indicating a complete inhibition. These findings are consistent to our earlier observations that reduced basal NO promotes activation of ICAM-1 via Src-mediated phosphorylation (Xu et al., 2013).

### Comparison of this study with studies using eNOS knock out animals

Genetic deletion of eNOS or over expression of caveolin-1 has shown increased leukocyte EC interaction, aggravated infection under disease conditions, and accelerated atherosclerosis in apolipoprotein E (apoE) deficient mice (Sasaki et al., 2003; Fernández-Hernando et al., 2009, 2010; Atochin and Huang, 2010; Fritzsche et al., 2010). Those studies indicated an important role of NO in maintaining vascular function and preventing vascular inflammation, but the mechanisms were not well defined. ApoE/eNOS double knock out mice show significant reduction of superoxide production, but increased leukocyte/EC interaction, macrophage infiltration in carotid arteries, and vascular cell adhesion molecular 1 (VCAM-1) expression in both endothelial and smooth muscle cells compared to apoE^−/−^ alone, suggesting a NO-dependent and ROS-independent mechanism to be involved in increased leukocyte/EC interaction (Ponnuswamy et al., 2012). The eNOS knockout animals basically represent a constantly reduced NO condition that may trigger some adaptation and compensatory effects in the vascular system. Therefore, the observed inflammatory manifestation is the results of an integration of multiple factors and signaling pathways. Our single vessel studies as that demonstrated in Figure 1 (Xu et al., 2013) enabled us to define the mechanisms involved in ECs, but has to rely on cultured cells or extracted proteins to provide further details. The main focus of our current study is to identify the mechanisms involved in a rapid EC response to reduced basal NO, which represents EC actions to acute or local NO production changes under physiological or pathological conditions, which could not be achieved using eNOS knockout animals. We are also cautious about the potential phenotype differences between cultured HUVECs and ECs in intact rat vessels. In this study, the basic results derived from cell culture studies have been consistent to our intact vessel findings. Importantly, the *in vitro* study enabled us to reveal the mechanisms at cellular and protein levels.

In Summary, ICAM-1 is constitutively expressed at low levels on ECs and it is indispensable for the normal functioning of the immune system (van de Stolpe and van der Saag, 1996), but its increased expression during inflammation or after stimulation by pro-inflammatory cytokines contributes to leukocyte/endothelial interaction and vascular inflammation (Rothlein et al., 1986; Granger and Kubes, 1994; Hubbard and Rothlein, 2000). On the other hand, constitutively expressed eNOS continuously produces a low level of NO and this basal NO acts as an endogenous inhibitor of leukocyte adhesion, activation and chemotaxis (Furchgott and Vanhoutte, 1989; Ignarro, 1989; Kubes et al., 1991; Moncada, 1992; Tsao et al., 1994). Despite accumulating evidence that ICAM-1 mediates adhesion and extravasation of leukocytes to and through the endothelium (Carlos and Harlan, 1994) and endogenous NO modulates leukocyte adherence through the expression of adhesion molecules on leukocyte and ECs (Kubes et al., 1991; Tsao et al., 1994), the underlying signaling mechanisms of reduced NO-mediated increases in adhesiveness of microvessel remained poorly understood. It has been proposed that eNOS-derived NO does not directly modulate EC conversion to a pro-adhesive phenotype but rather interferes with the generation of ROS by NADPH oxidase and prevents ROS-mediated inflammatory response (Niu et al., 1994; Kvietys and Granger, 2012). In contrast to this proposal, the timing and pattern of leukocyte adhesion in our previous study strongly supported a direct association between basal NO and adhesive states of ECs (Xu et al., 2013). Our present *in vitro* study on CAV-induced basal NO reduction directly demonstrated that basal NO is essential for maintaining the non-adhesive state of endothelium through regulation of the adhesive binding of endothelial ICAM-1. The reduced basal NO-induced increases in EC adhesive binding were mediated by Src-dependent ICAM-1 activation through its phosphorylated state. Although the potential structural changes of ICAM-1 that resulted in increased adhesion avidity following Src-mediated ICAM-1 activation remain to be elucidated, this study provides new insights into the mechanisms how reduced basal NO could mediate leukocyte adhesion to ECs. Importantly, these findings could be of greater importance not only in understanding the early pathologic stages of various clinical conditions where reduced endothelial-derived NO plays a central role in endothelial dysfunction, but also to the future therapeutic approaches in inflammation-induced endothelial dysfunction.

## Author contributions

FG and BL-W contributed equally for design, acquisition, analysis, draft MS. PH contributed to design, data analysis, interpretation of the work, MS writing. XL, AL, L-CX, and SX contributed to data acquisition, analysis, and MS draft. KL and MT contributed to data analysis and MS revision. CS, JH, and CR contributed to the data interpretation, and MS revision. All authors had final approval of the submitted version and agreement to be accountable for all aspect of the work.

### Conflict of interest statement

The authors declare that the research was conducted in the absence of any commercial or financial relationships that could be construed as a potential conflict of interest.
